# Positive effects of low-dose S-ketamine on preventing myocardial injury after thoracoscopic lobectomy in patients aged 70 to 85

**DOI:** 10.1186/s12871-024-02491-z

**Published:** 2024-03-19

**Authors:** Ziqiang Bi, Lijuan Kong, Jiahui Zhao, Dongdong Song, Fengmei Duan

**Affiliations:** Department of Anesthesiology, Affiliated Hospital of Chengde Medical College, 36 Nanyingzi Street, Chengde, 067020 Hebei Province China

**Keywords:** S-ketamine, Older patients, Pulmonary lobectomy, Myocardial injury, Goal-directed liquid treatment

## Abstract

**Objective:**

To investigate the effects of low-dose S-ketamine on marker of myocardial injury (BNP, hs-cTnT and HFABP) after thoracoscopic lobectomy in patients aged 70 to 85.

**Methods:**

One hundred patients (four cases excluded) aged 70–85 years, with body mass index 18–24 kg·m^−2^ and American Society of Anesthesiologists physical status II–III, scheduled for elective lobectomy from April 2022 to April 2023, were selected. The patients were divided into two groups by a random number table method, namely, the low-dose S-ketamine combined with GDFT group (group S) and the control group (group C), with 48 cases in each group. In group S, a low dose of S-ketamine (0.2 mg/kg) was given 1 min before intubation, and the maintenance dose was 0.12 mg·kg^−1^·h^−1^. Fluid therapy, guided by cardiac index (CI), changes in stroke volume (△SV), and other dynamic indicators, was used for rehydration during the operation. Group C was given the same amount of normal saline (0.2 mg/kg) 1 min before intubation, and the same rehydration therapy was adopted during the operation. The mean arterial pressure (MAP) and heart rate (HR) of the two groups were observed and recorded immediately after entering the operating room (T0), immediately after intubation (T1), immediately after the beginning of one-lung ventilation (OLV) (T2), immediately after the beginning of surgery (T3), immediately after the end of OLV (T4), and at the end of surgery (T5). The intraoperative fluid intake and output and the use of vasoactive drugs were recorded. The plasma levels of heart-type fatty acid–binding protein (HFABP), high-sensitivity troponin T (hs-cTnT), brain natriuretic peptide (BNP), interleukin-6 (IL-6), interleukin-8 (IL-8), and tumor necrosis factor-α (TNF-α) were recorded 24 h before operation and 24 and 48 h after operation. Visual analogue scale (VAS) pain scores at rest were recorded at 2 (V1), 6 (V2), 12 (V3), 24 (V4), and 48 h (V5) after operation, and the occurrence of myocardial ischemia during hospitalization was noted.

**Results:**

Compared with group C, MAP was significantly higher at T1–T5 in group S (*P* < 0.05), and the plasma concentrations of IL-6, IL-8, TNF-α, BNP, hs-cTnT, and HFABP were significantly lower at 24 and 48 h after operation (*P* < 0.05). The VAS pain scores at 2, 6, 12, 24, and 48 h after operation, the number of effective patient-controlled intravenous analgesia (PCIA) compressions, and the total number of PCIA compressions within 48 h after operation were significantly decreased (*P* < 0.05). Compared with group C, The hospitalization days, and the incidence of postoperative myocardial ischemia in group S were lower (*P* < 0.05). There were no significant intergroup differences in urine volume, extubation time, the incidence of postoperative atrial fibrillation, bleeding volume, colloid infusion volume, total fluid infusion volume, and the incidence of rescue analgesia.

**Conclusions:**

Low-dose S-ketamine can reduce the levels of hs-cTnT, HFABP, and BNP in older patients after pulmonary lobectomy, which has a positive effect on preventing myocardial injury.

**Trial registration:**

This study was registered on CHICTR (registration No. ChiCTR2300074475). Date of registration: 08/08/2023.

**Supplementary Information:**

The online version contains supplementary material available at 10.1186/s12871-024-02491-z.

## Introduction

Myocardial injury after noncardiac surgery (MINS) is an emerging pathological entity that often occurs within 48 h after surgery. Most patients fail to manifest typical symptoms because of analgesic drugs and mechanical ventilation. The diagnosis of MINS mainly depends on serological changes: a postoperative troponin T level of ≥ 30 ng/L or an increase in postoperative hypersensitive troponin T (hs-cTnT) level to 20–65 ng/L and change in an absolute value of ≥ 5 ng/L or an hs-cTnT level of ≥ 65 ng/L or change in an absolute value of ≥ 14 ng/L [1]. The current commonly used myocardial injury markers are troponin and BNP, but they have an obvious time lag in the diagnosis of acute myocardial infarction, so they cannot be detected before the occurrence of myocardial necrosis due to myocardial ischemia. Some studies have shown that HFABP and hs-cTnT are sensitive indicators for early myocardial injury [2, 3]. hs-cTnT is located in the myocardial fibers on unique regulatory proteins. When the myocardial cell membrane is intact, hs-cTnT cannot pass through the cell membrane, so it cannot be detected in the peripheral blood of healthy people. hs-cTnT is highly sensitive to myocardial injury and can appear in the early stage of myocardial injury. It can be detected even at a very low concentration, thereby improving the early diagnosis of myocardial injury [4]. BNP is regulated by left ventricular tone and is mainly produced and released by left ventricular cardiomyocytes. In myocardial injury, BNP may increase earlier than troponin, so it can be used as a sensitive index for the early diagnosis of myocardial injury caused by various factors [5]. HFABP is widely present in human cardiomyocytes and is rapidly released into the blood during myocardial ischemia and hypoxia. HFABP has high sensitivity and specificity in the diagnosis of early myocardial microinjury [6]. Due to the decline in physical function, surgical methods, mental stress, and other related factors, older patients often experience drastic fluctuations in the body's internal environment and circulatory stability during the perioperative period, which reduces the effective perfusion of their heart and causes myocardial cell damage. Therefore, perioperative sedation, analgesia, and fluid therapy are particularly important in older patients. Goal-directed fluid therapy (GDFT) can maintain perioperative tissue and organ perfusion and hemodynamic stability, thereby improving the perioperative prognosis of older patients [7]. S-ketamine is a new type of anesthetic drug with analgesic and sedative effects. Considering that its effects on the cardiovascular system are mainly mediated by effects on the sympathetic nervous system, its use has been encouraged for anesthesia in patients with hemodynamic instability [8]. Based on the analgesic intensity ratio of ketamine and S-ketamine, the optimal low dose of ketamine for intravenous injection has been found to be ≤ 0.5 mg/kg [9]. In older patients, the drug metabolism decreases due to organ dysfunction, the blood drug concentration is usually high, and the dose should be reduced as appropriate. The aim of this study was to investigate the effect of low-dose (0.2 mg/kg) S-ketamine on myocardial injury after pulmonary lobectomy in older patients.

## Methods/design

### Ethics, consent, and permissions

This study was registered on CHICTR (registration No. ChiCTR2300074475),date of registration: 08/08/2023. It was approved by the hospital’s ethics committee (CYFYLL2022455), and patients or their families signed informed consent. We selected 100 older patients with thoracoscopic lung resection (excluding four cases), male or female, 70–85 years old, with body mass index (BMI) 18–24 kg/m^2^, American Society of Anesthesiologists (ASA) grade II–III, and expected one-lung ventilation (OLV) time 1–2 h.

### Randomization and blinding

The participants were randomly allocated to the following groups: (1) the low-dose S-ketamine group (group S) and (2) the control group (group C). The patients were allocated at a 1:1 ratio using random numbers generated by Microsoft Excel. The specific method of random grouping was as follows: we entered a set of data from 1 to 100 in column A, entered the formula “ = RAND ()” in column B1, and entered dropdown formula from B1 to B96 to generate random data, arranging the data in column B in ascending order. The patients were numbered serially based on the order of admission, where the numbers above line A48 (including A48) were assigned to group S, whereas those below line A48 were assigned to group C. Electrocardiogram (ECG), blood oxygen saturation (SpO_2_), and noninvasive arterial blood pressure (NIBP) were monitored in both groups after admission, and radial artery puncture was performed under local anesthesia. The patients in group S were given a low dose of S-ketamine (0.2 mg/kg) 1 min before intubation, the maintenance dose was 0.12 mg·kg^−1^·h^−1^ during the operation, and the drug was stopped 30 min before the end of the operation. The cardiac index (CI), change in stroke volume (△SV), and other dynamic indicators monitored using the FloTrac/Vigileo system were used to guide fluid therapy. The patients in group C were given the same amount of normal saline (0.2 mg/kg) 1 min before intubation, and the same fluid therapy was adopted during the operation.

The intraoperative hemodynamic management plan was as follows. If CI was < 2.5 L·kg^−1^·min^−1^, 6% hydroxyethyl starch (200 mL) was rapidly infused within 10 min. If ΔSV was ≥ 10% and mean arterial pressure (MAP) was < 60 mm Hg, the administration was repeated until MAP was ≥ 60 mm Hg and this target value was maintained. If ΔSV was < 10%, inotropic drugs (0.2 mg dopamine each time) were administered. If CI was 2.5 L·kg^−1^·min^−1^ and MAP was < 60 mm Hg, 6% hydroxyethyl starch (200 mL) was rapidly infused within 10 min. If ΔSV was ≥ 10% and MAP was < 60 mm Hg, the administration was repeated until MAP was > 60 mm Hg. If ΔSV was < 10% and MAP < 60 mm Hg, vasoconstrictor drugs (4 μg norepinephrine) were given until MAP was ≥ 60 mm Hg.

### Study selection and inclusion criteria

Inclusion criteria were as follows:

- older patients (70–85 years old) scheduled to undergo elective thoracoscopic pulmonary lobectomy;
- expected OLV time of 1–2 h;
- BMI 18.5–24.0 kg/m^2^.

Exclusion criteria were as follows:

- a history of cardiogenic shock or myocardial infarction in the past 6 months;
- refusal to participate in this study;
- ASA grade > III;
- severe hypertension, heart disease, and other important organ dysfunction;
- history of smoking, alcohol use, or drug dependence.

### Interventions

The ECG monitoring system was established immediately after entering the room to monitor the heart rate (HR), blood pressure (BP), and SpO_2_. Anesthesia was induced with propofol (1.5–2.5 mg/kg), midazolam (0.05–0.2 mg/kg), cisatracurium (0.2 mg/kg), and sufentanil (0.8–1.0 μg/kg) in 15–20 s. Anesthesia was maintained by inhalation of 1.0 MAC sevoflurane, continuous intravenous infusion of remifentanil (0.1–0.2 μg·kg^−1^·min^−1^) and propofol (1.5 mg·kg^−1^·h^−1^). Approximately 3 min after oxygen denitrification, double-lumen endobronchial intubation was performed, and the position and depth of the catheter were confirmed by fiberoptic bronchoscopy. Bispectral index (BIS) was maintained between 40 and 60, and the fluctuation of blood pressure (BP) did not exceed 20% of the admission BP. During OLV, all of the patients were treated with volume-control ventilation: tidal volume (TV) = 6–8 mL/kg, respiratory rate (RR) = 12–15 times/min, FiO_2_ 60%, and inhalation: exhalation (I:E) ratio = 1:2, SpO_2_ > 90% and P_et_CO_2_ = 35–45 mm Hg were maintained by adjusting I:E ratio and FiO_2_ during operation. Inhaled anesthetics were stopped when a subcutaneous suture was performed, and propofol and remifentanil were continued to maintain anesthesia until the end of the skin suture. All of the patients received patient-controlled intravenous analgesia (PCIA) with sufentanil 200 μg, butorphanol 8 mg, palonosetron 0.25 mg, and normal saline 300 mL, with background dose of 3 mL/h, patient-controlled intravenous single dose of 3 mL, and lockout time of 30 min. When the visual analogue scale (VAS) pain score at rest was ≥ 5, a single intravenous injection of sufentanil 5 μg was given as needed for rescue analgesia.

### Observation index and detection method

The anesthesia time, operation time, OLV time, HR, and MAP were recorded immediately after entering the operating room (T0), immediately after intubation (T1), immediately after the beginning of OLV (T2), immediately after the beginning of surgery (T3), immediately after the end of OLV (T4), and at the end of surgery (T5). We also recorded the amount of colloid infusion, total amount of fluid, blood loss, urine volume, and use of norepinephrine, dopamine, and escitalopram ketamine. Peripheral venous blood (5 mL) was collected 24 h before the operation and 24 and 48 h after the operation, and plasma was separated by centrifugation. The plasma level of heart-type fatty acid binding protein (HFABP) was measured by double-antibody sandwich enzyme-linked immunosorbent assay. The levels of high-sensitivity troponin T (hs-cTnT), brain natriuretic peptide (BNP), interleukin-6 (IL-6), interleukin-8 (IL-8), and tumor necrosis factor-α (TNF-α) were measured. The resting VAS pain scores at 2 (V1), 6 (V2), 12 (V3), 24 (V4), and 48 h (V5) after the operation extubation time, arrhythmias(postoperative atrial fibrillation), hospitalization days and postoperative myocardial ischemia were recorded. (The diagnosis of Myocardial ischemia mainly depends on changes: 1. chest discomfort, arm discomfort, neck discomfort, jaw discomfort, shortness of breath, or pulmonary edema. These ischemic symptoms had to have occurred within 24 h of an elevated hsTnT measurement. 2. Ischemic electrocardiography findings included any of the following: i. development of pathologic Q waves in any two contiguous leads that were ≥ 30 ms; ii. development of left bundle branch block (LBBB); or iii. development of ST segment elevation (≥ 2 mm in leads V1, V2, or V3 OR ≥ 1 mm in the other leads), ST segment depression (≥ 1 mm), or symmetric inversion of T waves ≥ 1 mm in at least two contiguous leads. ST segment elevation, ST segment depression, and LBBB had to have occurred within 3 days of an elevated hsTnT measurement, and symmetric T wave inversion had to have occurred within 5 days of an elevated hsTnT measurement. 3. New or presumed new wall motion abnormality on echocardiography. 4. New or presumed new fixed defect on radionuclide imaging [10]).

### Statistical analysis

The required sample size was calculated using PASS 23.0 software. The results of the pilot test showed that hs-cTnT was 21.3 ± 1.7 ng/L in group S and 25.4 ± 1.5 ng/L in group C at 24 h after operation (*P* < 0.05). With α = 0.05 (two-sided test) and 1 − β = 0.9, a total of 40 patients in each group had to be included, and the dropout rate was expected to be 20%. In order to improve the test power, 50 patients were planned to be included in each group.

SPSS 25.0 software was used for the statistical analysis. Measurement data with normal distribution were expressed as mean ± standard deviation (*x* ± *s*). *T-*test was used for intergroup and repeated measures ANOVA was used for intragroup comparisons. Count data were expressed as number (%), and intergroup comparisons were done using the chi-square test. A *P* value < 0.05 was considered to be statistically significant.

## Results

### Search results and study characteristics

**Fig. 1:**
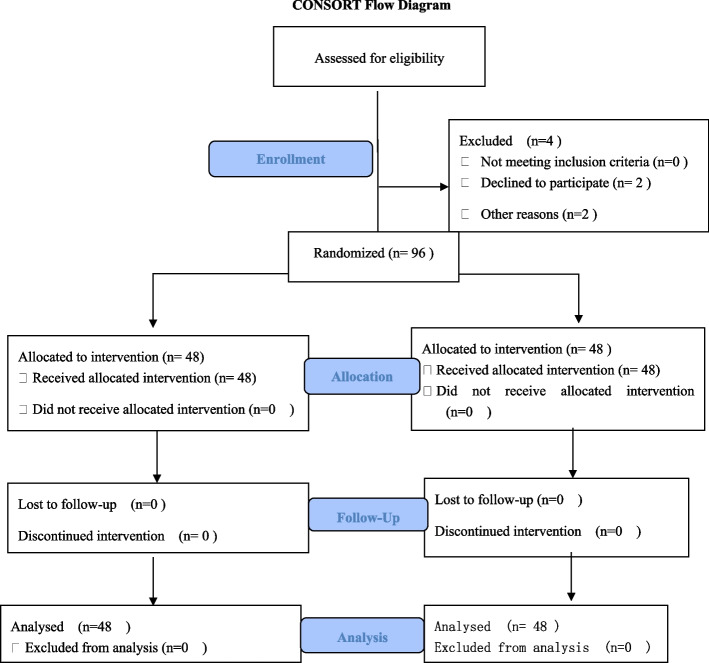
CONSORT flow diagram

A total of 100 patients were selected for this study and four patients were excluded, resulting in a total of 48 patients in each of the two groups. A CONSORT diagram showing the flow of participant recruitment throughout each stage of the randomized trial is shown in (Fig. 1).

There were no significant intergroup differences in gender, age, BMI, ASA grade, OLV time, anesthesia time, and operation time. Compared with group C, the amount of colloid infusion, the total amount of fluid infusion, the use of norepinephrine postoperative myocardial ischemia, and dopamine were significantly lower in group S (*P* < 0.05). Compared with group C, the hospitalization days of group S (*P* < 0.05) is shorter. There were no significant differences between urine volume, extubation time, postoperative atrial fibrillation and blood loss (Table 1).

**Table 1:** Comparison of general conditions between the two groups

	**S-ketamine group (*N* = 48)**	**Control group (*N* = 48)**	**P**
Male/Female	**25/23**	**24/24**	** > 0.99**
Age(year)	**78.38 ± 1.36**	**78.20 ± 1.31**	**0.50**
BMI(kg/m^2^)	**21.97 ± 1.14**	**21.96 ± 1.27**	**0.97**
ASA II/III	**21/27**	**22/26**	** > 0.99**
Weight(kg)	**60.26 ± 3.53**	**60.38 ± 5.41**	**0.89**
Height(m)	**1.65 ± 0.05**	**1.66 ± 0.06**	**0.65**
Anesthesia time (min)	**87.46 ± 2.52**	**87.90 ± 2.54**	**0.38**
Time of operation(min)	**75.28 ± 1.99**	**75.08 ± 1.63**	**0.58**
Time of OLV(min)	**57.08 ± 2.97**	**56.18 ± 2.77**	**0.12**
Colloid volume (ml)	**434.20 ± 3.63**	**461.92 ± 13.23**	** < 0.001**
Total fluid infusion volume(ml)	**1099.00 ± 202.15**	**1325.60 ± 181.38**	** < 0.001**
Blood loss (ml)	**143.84 ± 18.17**	**145.83 ± 18.15**	**0.58**
Urine volume (ml)	**442.04 ± 2.62**	**443.02 ± 2.67**	**0.06**
Norepinephrine adrenal gland [case (times)]	**5 (8)**	**14(17)**	**0.02**
Dopamine [case (times)]	**4 (7)**	**12(14)**	**0.03**
Myocardial ischemia[ case(%)]	**3(6)**	**10(20)**	**0.04**
Postoperative atrial fibrillation [ case(%)]	**5(10)**	**9(18)**	**0.24**
Extubation time(min)	**12.31 ± 3.12**	**12.52 ± 2.94**	**0.73**
Hospitalization (days)	**7.67 ± 0.47**	**8.89 ± 0.78**	** < 0.001**

Data were presented as mean ± SD or counts

*ASA* American society of anesthesiologists, *BMI* body mass index, *OLV* one lung ventilation

**Fig. 2:**
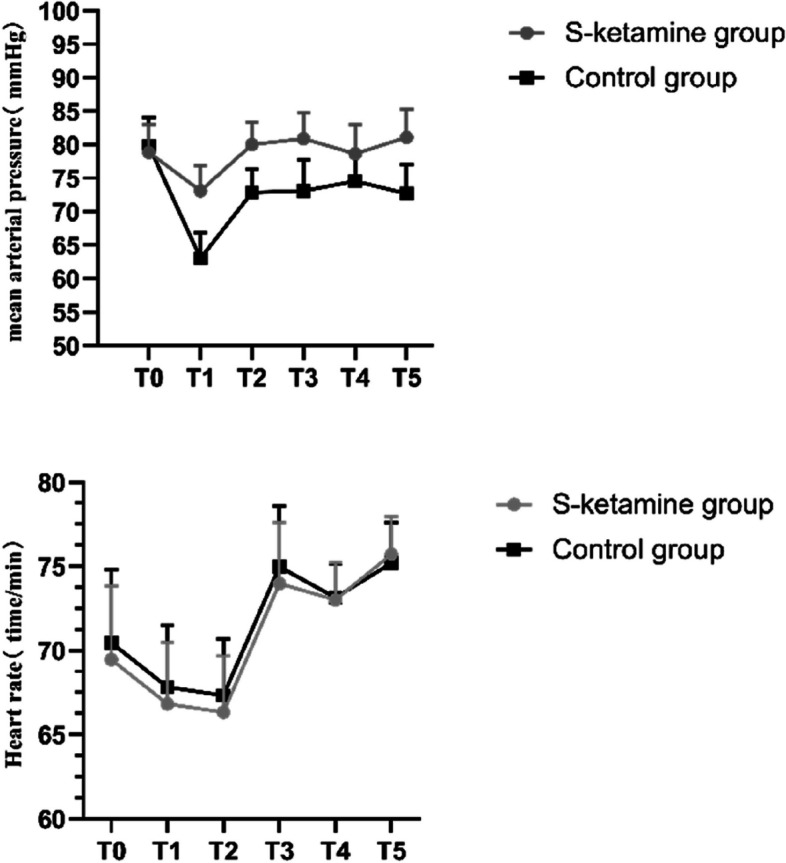
Comparison of blood pressure and heart rate between the two groups

**Fig. 3:**
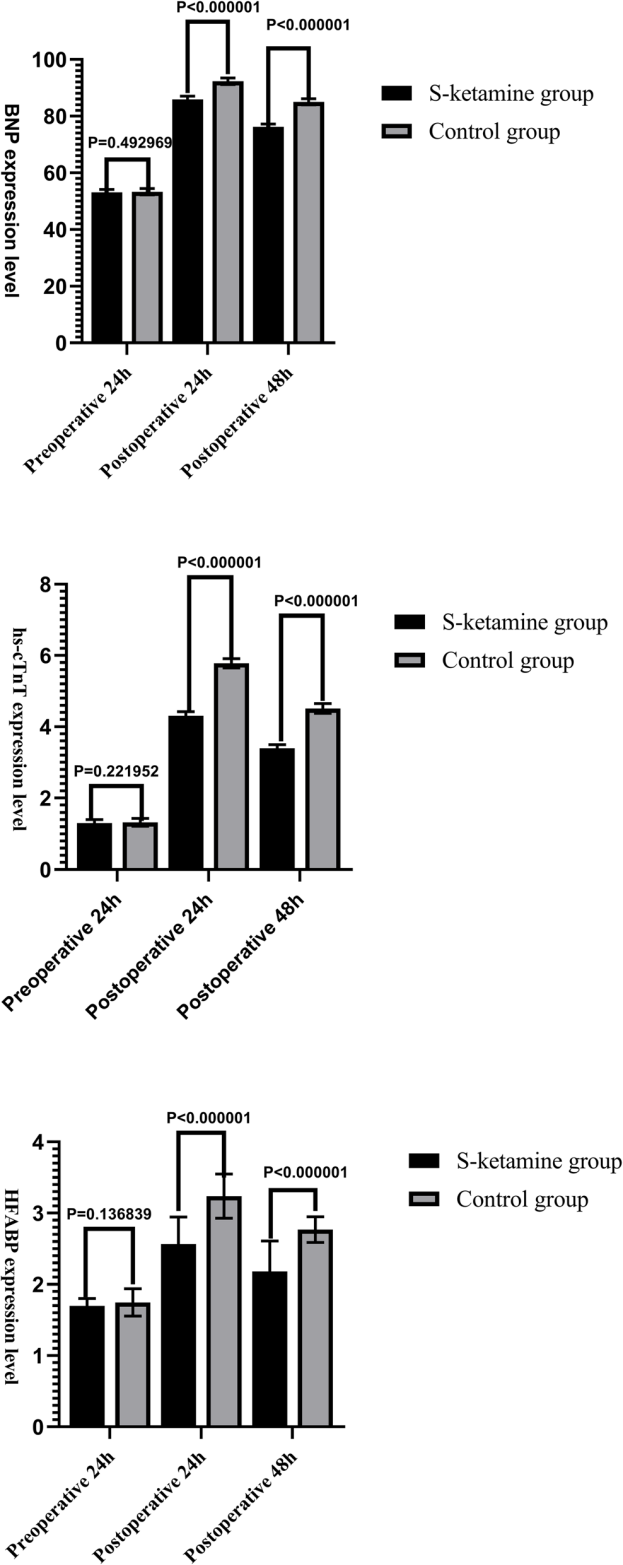
Comparison of markers of myocardial injury between the two groups

Compared with group C, T1–T5 MAP was significantly increased in group S (*P* < 0.05). There were no significant intergroup differences in HR at T0–T5 and MAP at T0 (*P* > 0.05). The comparison of mean arterial pressure and heart rate at different time points within each group was statistically significant (*P* < 0.05) (Table 2 or Fig. 2). Compared with group C, the plasma levels of BNP, hs-cTnT, and HFABP at 24 and 48 h after operation were significantly lower in group S (*P* < 0.05). The comparison of the plasma levels of BNP, hs-cTnT, and HFABP at different time points within each group was statistically significant (*P* < 0.05). (Fig. 3).

**Table 2:** Blood pressure and heart rate were compared between the two groups

	Group	T_0_	T_1_	T_2_	T_3_	T_4_	T_5_
**HR(time/min)**	**S-ketamine group** **(*N* = 48)**	**69.68 ± 4.52**	**66.72 ± 3.67**	**66.48 ± 3.44**	**73.91 ± 3.58**	**72.90 ± 2.30**	**75.58 ± 2.42**
	**Control group** **(*N* = 48)**	**70.68 ± 4.50**	**67.70 ± 3.65**	**67.48 ± 3.45**	**74.93 ± 3.60**	**73.10 ± 2.03**	**75.06 ± 2.49**
	**P**	**0.27**	**0.18**	**0.15**	**0.17**	**0.65**	**0.29**
**MAP(mmHg)**	**S-ketamine group** **(*N* = 48)**	**88.98 ± 4.41**	**83.10 ± 4.17**	**89.98 ± 3.56**	**90.52 ± 4.23**	**88.24 ± 4.68**	**90.86 ± 4.43**
	**Control group** **(*N* = 48)**	**89.84 ± 4.56**	**73.14 ± 3.87**	**82.96 ± 3.55**	**82.85 ± 4.68**	**84.22 ± 4.64**	**82.83 ± 4.42**
	**P**	**0.34**	** < 0.001**	** < 0.001**	** < 0.001**	** < 0.001**	** < 0.001**

Data were presented as mean ± SD or counts

*HR* Heart rate, *MAP* Mean artery pressure

**Fig. 4:**
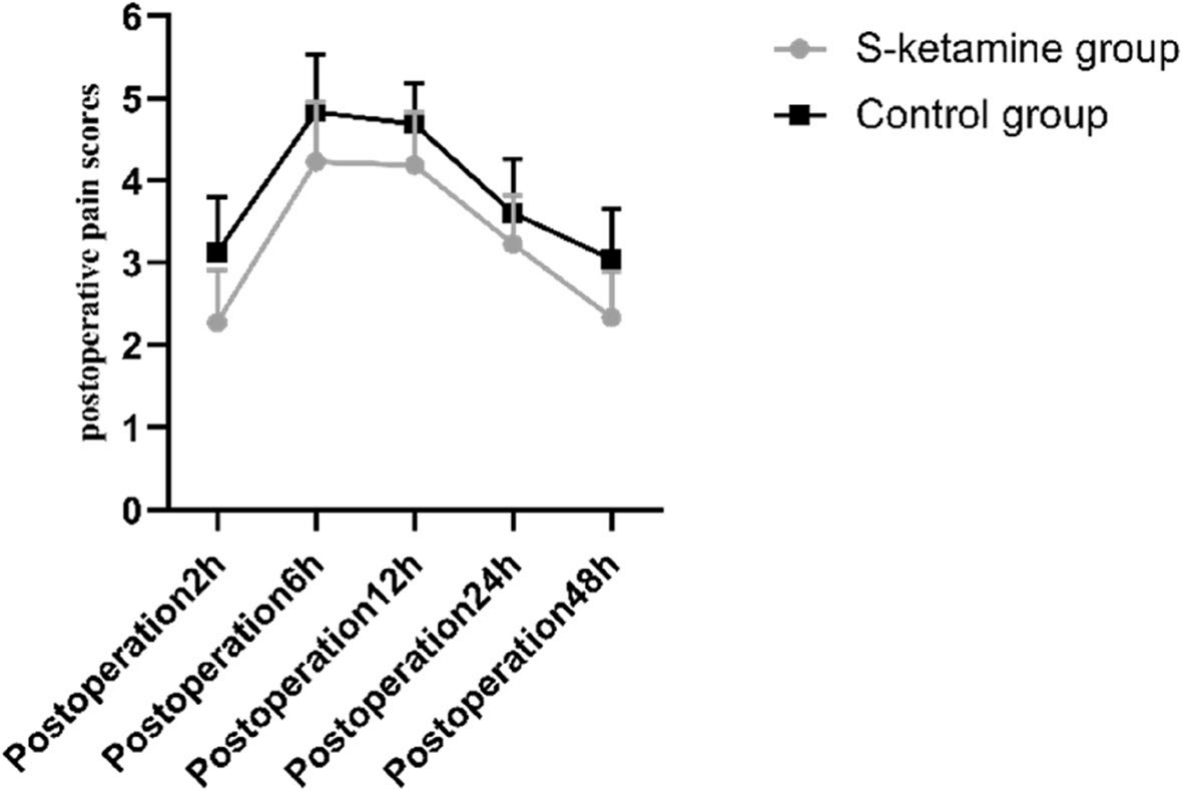
Comparison of the postoperative pain scores between the two groups

Compared with group C, VAS pain scores at rest at 2, 6, 12, 24, and 48 h after operation, the number of effective PCIA compressions, and the total number of PCIA compressions within 48 h after operation were significantly lower in group S (*P* < 0.05). There was no significant intergroup difference in the incidence of rescue analgesia. The comparison of VAS pain scores at different time points within each group was statistically significant (*P* < 0.05) (Table 3 or Fig. 4).

**Table 3:** The postoperative pain scores of the two groups were compared

	**S-ketamine group (*N* = 48)**	**Control group (*N* = 48)**	**P**
**Postoperation2h**	**2.28 ± 0.64**	**3.14 ± 0.67**	** < 0.001**
**Postoperation6h**	**4.24 ± 0.72**	**4.84 ± 0.69**	** < 0.001**
**Postoperation12h**	**4.18 ± 0.63**	**4.70 ± 0.49**	** < 0.001**
**Postoperation24h**	**3.22 ± 0.58**	**3.60 ± 0.65**	** < 0.001**
**Postoperation48h**	**2.34 ± 0.56**	**3.02 ± 0.62**	** < 0.001**
**PCIA effective pressure(time)**	**2.98 ± 0.55**	**3.42 ± 0.62**	** < 0.001**
**PCIA total pressure(time)**	**3.70 ± 1.11**	**4.74 ± 0.78**	** < 0.001**
**Rescue analgesia [case (%)]**	**6(13)**	**8(17)**	**0.56**

Data were presented as mean ± SD or counts

*PCIA* Patient-controlled intravenous analgesia

**Fig. 5:**
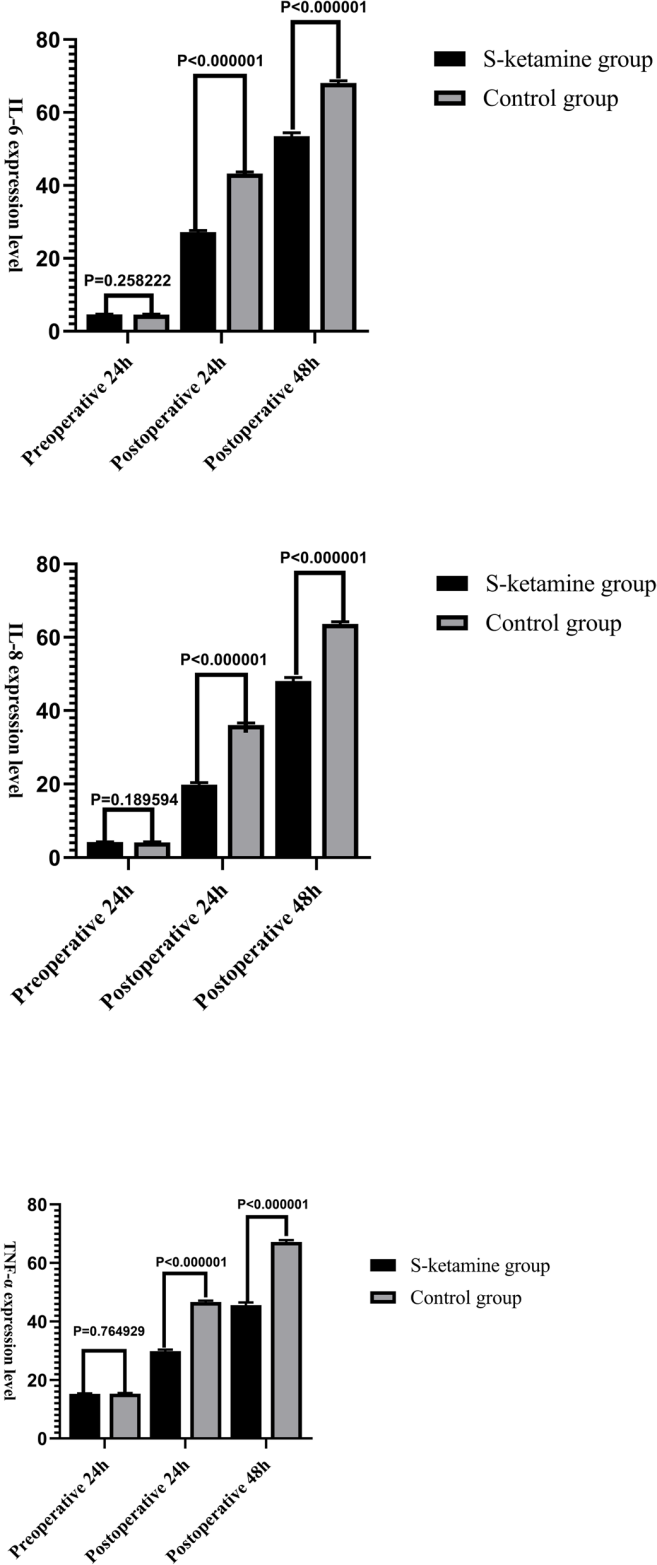
Comparison of inflammatory factors between the two groups

Compared with group C, there was no significant difference in IL-6, IL-8, and TNF-α at 24 h before operation in group S, and the levels of IL-6, IL-8, and TNF-α were significantly lower at 24 and 48 h after operation (*P* < 0.05) (Fig. 5).

## Discussion

Perioperative fluid infusion is one of the important processes of anesthesia management. A recent study found that there is a "U"-shaped relationship between postoperative complications and perioperative intravenous fluid volume [11]. Excessive fluid infusion may induce complications such as tissue edema and cardiac overload, while insufficient fluid infusion may induce complications related to hypovolemia. A previous study confirmed that GDFT can provide individualized rehydration therapy for patients by monitoring hemodynamics or tissue perfusion, which can effectively avoid potential excessive or insufficient blood volume and is an ideal fluid control measure [12]. GDFT can guide fluid resuscitation quantitatively under dynamic monitoring indicators, which can effectively maintain cardiac load and blood circulation volume and reduce the occurrence of heart failure and myocardial infarction, preventing cardiovascular and cerebrovascular complications [13] and reducing postoperative mortality [14].

MINS occurs secondary to myocardial ischemia caused by a mismatch between oxygen supply and demand, or thrombosis, which may lead to myocardial necrosis and have prognostic relevance [15]. In recent years, an increasing number of studies have focused on MINS but the severity of its adverse consequences is not yet fully recognized [10, 16]. As a special group, older patients have poor tolerance to major surgery, especially because they have poor myocardial compliance and unstable coronary hemodynamics, which affect myocardial perfusion and oxygen supply, and are susceptible to myocardial injury. The results of this study showed that compared with the control group, the plasma concentrations of BNP, hs-cTnT, and HFABP of the patients in the low-dose S-ketamine combined with GDFT group were significantly lower at 24 and 48 h after surgery (*P* < 0.05), which is consistent with the results of Mazzeffi et al. and Ishimaru et al. [17, 18]. Hence, low-dose S-ketamine can maintain hemodynamic stability during anesthesia; ensure effective perfusion of important organs, such as the heart; and reduce myocardial cell injury in patients undergoing noncardiac surgery. It can also reduce the concentrations of BNP, hs-cTnT, and HFABP in the blood, and it has a protective effect on myocardial cells.

S-ketamine, a chiral cyclohexanone derivative isolated from ketamine, is a newly marketed intravenous anesthetic in China. It has the advantages of rapid drug metabolism, short recovery time, and few adverse reactions. The results of this study showed that there was no significant difference in MAP between the patients in the low-dose S-ketamine combined with GDFT group and the patients in the control group immediately after entering the operating room. At the end of the immediate intubation to surgery, MAP in group S was higher than that in the control group. The low-dose S-ketamine combined with GDFT group showed significantly lower use of vasoactive drugs than the control group, which is consistent with the results of Wang et al. [19] and Yang et al. [20]. This means that the application of a low dose of S-ketamine during operation can activate the sympathetic nervous system, increase blood pressure and cardiac output, reduce the use of anesthetic drugs [21], and help maintain hemodynamic stability in older patients [20].

The myocardioprotective effect of S-ketamine may be related to its ability to activate the nuclear factor-erythroid 2-related factor 2/heme oxygenase-1 signaling pathway to enhance its endogenous antioxidant protection mechanisms, and it may also be related to its ability to inhibit necrotizing apoptosis and trigger related inflammatory pathways. A previous study has shown that the postoperative level of inflammatory factors is directly related to the degree of myocardial injury in elderly patients [22]. Production and release of inflammatory cytokines such as TNF-α, IL-6, and IL-8 promote neutrophil activation and their aggregation and adhesion to endothelial cells caused by the body's vascular endothelial barrier dysfunction, which leads to tissue edema and increased tissue damage; at the same time, the large number of neutrophils increases the permeability of myocardial cells, causing myocardial cell dysfunction and degeneration necrosis [23]. TNF-α, which is mainly produced by macrophages, is a multifunctional cytokine that is released in the early stage of systemic inflammatory response and is also the initiator of the release of other inflammatory factors, so it can reflect the severity of noxious stimulation to a certain extent. IL-6 and IL-8 are proinflammatory cytokines that are mainly secreted in the acute phase, so they may be reliable and sensitive biomarkers of inflammation [24]. In this study, according to the results of the low-dose S-ketamine combined with GDFT group compared with the control group, the levels of IL-6, IL-8, and TNF-α at 24 and 48 h after surgery were significantly lower, which is consistent with the results of Tu et al. [25]. These findings indicate that low-dose S-ketamine has a positive anti-inflammatory effect, effectively inhibits the production of inflammatory factors, and reduces the inflammatory response of the body, thereby improving the occurrence of postoperative myocardial injury in patients undergoing noncardiac surgery; thus, it may be more conducive to the early postoperative rehabilitation of patients [26, 27].

The new NMDA-receptor antagonist, S-ketamine, has an analgesic effect, prolongs postoperative analgesia time, and reduces postoperative analgesic drug dosage by inhibiting central sensitization [28]. The results of this study showed that compared with the patients in the control group, the patients in the low-dose S-ketamine combined with GDFT group had significantly lower VAS pain scores at different time points after surgery and significantly lower use of PCIA. The results are consistent with those of Ohtaki et al. [29]. This suggests that intravenous low-dose S-ketamine can reduce postoperative pain score and improve the effect of postoperative analgesia. There was no significant difference in the frequency of rescue analgesia between the two groups, which may be due to the better analgesic effect of the PCIA regimen, but this aspect is worthy of further exploration.

There are some shortcomings to this study, namely, the number of subjects was relatively small, the observation time was limited, the long-term survival rate of the patients was not recorded, and the detection indicators were limited. Therefore, additional rigorously designed multicenter large-sample clinical trials are needed to confirm whether low-dose S-ketamine offers long-term benefits for older patients undergoing lung resection.

## Conclusion

In conclusion, low-dose S-ketamine can reduce the plasma concentrations of BNP, hs-cTnT, HFABP, IL-6, IL-8, and TNF-α in older patients after pulmonary lobectomy. It has a positive effect on preventing myocardial injury after pulmonary lobectomy in older patients.

## Dissemination policy

The results will be communicated with anesthesiologists and thoracic surgeons via publications and academic conferences.

### Supplementary Information

**Supplementary Material 1.**

## Data Availability

All data generated or analysed during this study are included in this published article [and its Supplementary information files].
